# Associations between Carotid Artery Plaque Score, Carotid Hemodynamics and Coronary Heart Disease

**DOI:** 10.3390/ijerph121114275

**Published:** 2015-11-09

**Authors:** Huiping Zhang, Mengxue Liu, Tiantian Ren, Xiangqian Wang, Dandan Liu, Mingliang Xu, LingFei Han, Zewei Wu, Haibo Li, Yu Zhu, Yufeng Wen, Wenjie Sun

**Affiliations:** 1 Department of Medical Ultrasonics, Ma’anshan People’s Hospital, Ma’anshan 243000, China; E-Mails: zhhp1216@sina.com (H.Z.); rtt7219@sina.com (T.R.); wangxq0314@sina.com (X.W.); ldd1020@sina.com (D.L.); xml7498@sina.com (M.X.); 2 School of Public Health, Wannan Medical College, Wuhu 241000, China; E-Mails: lmxsnow11@sina.com (M.L.); wzwwnmc@sina.com (Z.W.); haiboli89@gmali.com (H.L.); kutuomonk@foxmail.com (Y.Z.); 3 Center of Clinical Laboratory, Ma’anshan Medical Group, Ma’anshan 243000, China; E-Mail: e76402664@sina.com; 4 School of Food Science, Guangdong Pharmaceutical University, Zhongshan 528458, China; 5 School of Public Health and Tropical Medicine, Tulane University, New Orleans, LA 70112, USA

**Keywords:** carotid intima-media thickness, carotid ultrasonography, coronary angiograph

## Abstract

*Background*: The carotid artery plaque score (PS) is an independent predictor of Coronary Heart Disease (CHD). This study aims to evaluate the combination of PS and carotid hemodynamics to predict CHD. *Methods*: A total of 476 patients who underwent carotid ultrasonography and coronary angiography were divided into two groups depending on the presence of CHD. PS, carotid intima-media thickness, and carotid blood flow were measured. Receiver operating characteristic curve analysis was performed to establish the best prediction model for CHD presence. *Results*: Age, sex, carotid intima-media thickness of internal carotid artery and carotid bifurcation, PS, peak systolic velocity (PSA) of right internal carotid artery (RICA), and most resistance index data were significantly related with the presence of CHD. The area under the curve for a collective model, which included factors of the PS, carotid hemodynamics and age, was significantly higher than the other model. Age, PS, and PSA of RICA were significant contributors for predicting CHD presence. *Conclusions*: The model of PS and PSA of RICA has greater predictive value for CHD than PS alone. Adding age to PS and PSA of RICA further improves predictive value over PS alone.

## 1. Introduction

In recent decades, the incidence and mortality rates of coronary heart disease (CHD) have increased in China, particularly in urban areas [1,2]. The absolute numbers of CHD events and deaths are predicted to increase dramatically between 2010 and 2029 [3]. The causes of atherosclerosis are multifactorial and identification of these factors could allow for earlier detection and prevention of CHD. Commonly used risk prediction factors, such as the Framingham risk score [4], have proven useful in identifying individuals at risk for CHD, but it can overestimate (or underestimate) risk in populations other than the U.S. population [5]. Plaque thickness predicts cardiovascular risk [6]. It is likely for that reason that intima-media thickness (IMT) studies that include plaque thickness predict cardiovascular risk [7], particularly in the elderly. However, IMT measured according to the Mannheim consensus does not represent atherosclerosis [8]; it is another phenotype [9]. Meta-analyses also found that IMT measured without plaque is a weak predictor of cardiovascular risk [10], and progression of IMT did not predict cardiovascular risk [11], nor did regression of IMT [12]. In the 7-year follow up report of that study, IMT in the common carotid did not predict coronary risk, IMT in the carotid bulb (including plaque thickness) was a weak predictor, and total plaque area was a strong predictor of coronary risk [13].

The carotid artery plaque score (PS) predicts CHD risk [14,15,16], and has been shown to be associated with CHD independent of C-IMT measurements [17]. However, it has not been adequately evaluated in risk classifications, especially using contemporary criteria for evaluating novel cardiovascular risk markers [18]. Recent studies show that carotid hemodynamics and vessel geometry play an important role in the cause of plaque formation [19,20,21]. Carotid blood flow can be used to assess carotid hemodynamics and vessel geometry [22]. We sought to assess whether a collective model, which includes PS and carotid artery blood flow, may have greater predictive power for identifying CHD than PS alone.

## 2. Methods

### 2.1. Patient Population and Selection

This was a cross-sectional study. Subjects were patients admitted to Ma’anshan People’s Hospital from December 2013 to June 2014. There 476 CHD patients who underwent carotid ultrasonography and coronary angiography simultaneously were enrolled this study. It was a consecutive collection of enrolled patients. Inclusion criteria: all patients without congenital heart disease, rheumatic heart disease, hypertensive heart disease, pulmonary heart disease and primary cardiomyopathy. The patients were divided into two groups depending on CHD presence or absence. The Institutional Review board of the hospital approved the study and all patients provided informed consent (The Medical Ethics Committee of Ma’anshan People’s Hospital, No. 2014001). The risk factor variables evaluated in the present study included age, sex, hypertension, hyperlipidemia, diabetes mellitus (DM), and smoking status. Height and weight were measured and BMI was calculated.

### 2.2. Carotid Ultrasonography

The near and far walls of the common carotid artery (CCA) and the internal carotid artery (ICA) and carotid artery bifurcation (CAB) on both sides were scanned for hemodynamics with 5–13 MHz linear-array probes (Aloka-а7 and Aloka-а10, Tokyo, Japan; Philips-IU22, Columbia, FL, USA). Patients were examined in the supine position with the head slightly turned. According to current guidelines, standardized C-IMT was measured on the far wall of both CCAs on a 10 mm segment located 2 cm upstream from flow divider [23]. Carotid artery plaques were considered as focal wall thickening ≥ 1200 μm protruding into the arterial lumen [24]. Plaques were looked for on the near and far walls in all the above-described arterial segments by transversal and longitudinal scanning.

Carotid artery plaque score (PS) [25] was calculated by summing each single plaque with its maximum thickness in the ipsilateral carotid artery without considering the length of each plaque so as to obtain the plaque score of this side. Adding bilateral carotid plaque scores to obtain a total PS was then performed.

### 2.3. Coronary Angiography

Coronary angiography was performed with standard Judkins technique [26] by cardiologists who were blinded to the ultrasonography results. The functional significance of CHD was evaluated using the Gensini score [27].

## 3. Statistical Analysis

Statistical analyses were conducted with SPSS18.0. Descriptive statistics were expressed as mean ± SD for continuous variables and percentage for categorical variables. Differences in demographic characteristics and laboratory assay data were compared with the analysis of variance and Chi-square test. Multiple logistic regression analysis was used to determine the most relevant factors predicting the presence of CHD. Using CHD as the dependent variable (patients with CHD were recorded “1”, patients of control group were “0”) and used the age, sex, carotid intima-media thickness (internal carotid artery and carotid bifurcation), PS, peak systolic velocity of right internal carotid artery, and resistance index data as independent variables (sls = 0.05, els = 0.05). Receiver operating characteristic (ROC) curve analysis was performed to establish the best prediction model for the presence of CHD.

## 4. Results

### 4.1. Demographic Characteristics and Clinical Parameters between CHD and Non-CHD Groups

Table 1 shows that except for age and sex, other characteristics were not significantly different between groups. Regarding ultrasound parameters, IMT of CAB, PS and most RI data were significantly higher in CHD group than in the non-CHD group.

**Table 1:** Comparison of demographic characteristics and clinical parameters between CHD and non-CHD patients.

Characteristics			CHD (*n* = 321)	Non-CHD (*n* = 155)	*x*^2^/*t*	*P*-Value
Demographics						
Age(years)	45–59 years		57 (18.51%)	37 (25.69%)	4.88	0.027
	60–74 years		172 (55.84%)	81 (56.25%)		
	75–89 years		79 (25.65%)	26 (18.06%)		
Sex	male		180 (57.14%)	62 (41.89%)	9.38	0.002
	female		135 (42.86%)	86 (58.11%)		
BMI			19.64 ± 3.00	20.11 ± 2.87	1.12	0.266
Register	rural		194 (61.59%)	87 (58.78%)	0.33	0.490
	urban		121 (38.41%)	61 (41.22%)		
Education	under middle school		214 (67.94%)	98 (66.22%)	0.14	0.712
	middle school		62 (19.68%)	31 (20.95%)		
	beyond middle school		39 (12.38%)	19 (12.84%)		
Smoking	Yes		46 (31.08%)	14 (19.44%)	2.02	0.069
Hypertension	Yes		107 (72.30%)	48 (66.67%)	0.74	0.390
Hyperlipidemia	Yes		15 (10.14%)	4 (5.56%)	2.88	0.237
DM	Yes		33 (22.30%)	24 (33.33%)	3.07	0.080
TC			4.05 ± 0.92	4.19 ± 0.92	1.03	0.304
TG			1.46 ± 0.85	1.22 ± 0.56	1.12	0.264
LDL			2.41 ± 3.00	2.42 ± 0.66	0.05	0.970
Apo a			1.44 ± 0.21	1.22 ± 0.21	−0.51	0.614
Apo b			1.01 ± 0.24	0.84 ± 0.22	−0.5	0.612
Hcy			14.78 ± 11.58	12.74 ± 5.72	−1.25	0.199
Ultrasound Parameters						
IMT (mm)	CCA		0.94 ± 0.14	0.89 ± 0.20	−1.05	0.326
	ICA		0.83 ± 0.31	0.77 ± 0.16	−2.14	0.033
	CAB		1.65 ± 0.82	1.31 ± 0.60	−3.83	0.000
PS			2.79 ± 2.37	1.57 ± 2.46	−4.93	0.000
Peak systolic velocity (PSV)	CCA	left	65.28 ± 18.98	64.63 ± 18.20	−0.38	0.701
		right	63.14 ± 18.98	65.23 ± 18.20	1.40	0.250
	ICA	left	52.17 ± 16.00	51.06 ± 14.92	−0.49	0.644
		right	53.64 ± 18.98	49.93 ± 18.20	−2.08	0.017
End diastolic velocity (EDV)	CCA	left	17.55 ± 6.08	18.43 ± 6.98	2.40	0.464
		right	16.84 ± 18.98	17.77 ± 18.20	1.40	0.159
	ICA	left	19.69 ± 6.44	19.12 ± 7.02	−0.60	0.553
		right	19.28 ± 18.98	18.63 ± 18.20	−1.07	0.227
Resistance index (RI)	CCA	left	0.73 ± 0.06	0.71 ± 0.06	−2.57	0.010
		right	0.73 ± 0.06	0.71 ± 0.06	−2.16	0.032
	ICA	left	0.62 ± 0.07	0.61 ± 0.06	−1.12	0.263
		right	0.64 ± 0.07	0.61 ± 0.06	2.40	0.001

Data are means ± S.D. or *n* (%).

### 4.2. Multiple Logistic Analysis for CHD

Logistic regression analysis found that age (*OR* = 1.104, *P* = 0.02), PSV of right ICA (*OR* = 1.021, *P* = 0.04), and PS (*OR* = 1.566, *P* < 0.01) were significant predictors for the presence of CHD. The results are shown in Table 2.

**Table 2:** Multiple Logistic Analysis for CHD.

Factors	*β*	*B*	*S.E.*	*χ*^2^	*P*-Value	*OR* (95% *CI*)
Age	0.04	0.22	0.29	5.81	0.02	1.104 (1.008, 1.078)
PSV of right ICA	0.02	0.21	0.01	4.28	0.04	1.021 (1.001, 1.041)
PS	0.47	0.57	0.11	17.99	<0.01	1.566 (1.273, 1.926)

*β*: Regression coefficient; *B*: standardized regression coefficient; *S.E*: standard deviation; *χ*^2^: chi-square; *OR*: Odds Ratio; *CI*: Confidence Interval; PSV: peak systolic velocity; ICA: internal carotid artery; PS: carotid artery plaque score.

### 4.3. ROC Prediction Curve of Each Index of CHD

**Figure 1:**
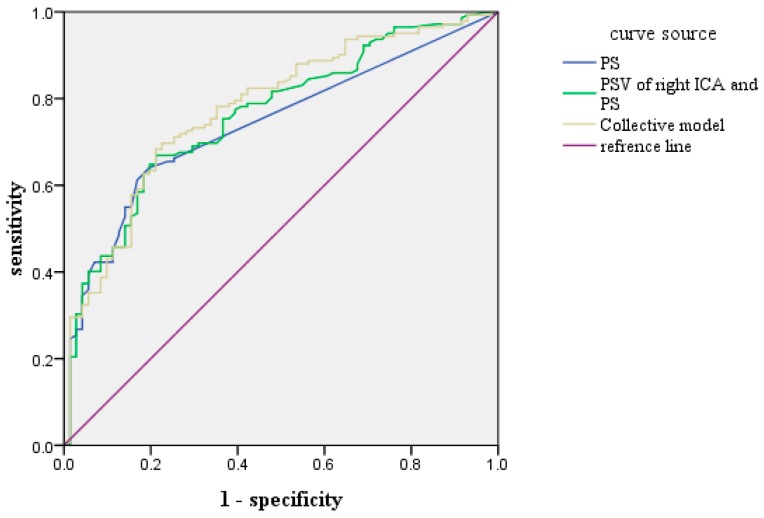
Receiver operating characteristic curve for the collective model. Collective model: including carotid ultrasonography (PSV of right ICA, PS) and age; PS: Carotid artery plaque score. AUC was obtained by receiver operating characteristic curve of the collective model. AUC: 0.778; sensitivity: 0.782; specificity: 0.648. In addition, AUC was also obtained by receiver operating characteristic curve of PS. AUC: 0.740; sensitivity: 0.613; specificity: 0.831.

Each index predicting the presence of CHD, was determined from the receiver operating characteristic curve. The results are shown in Table 3 and Figure 1. The AUC of the collective model of carotid ultrasonography (PSV of right ICA, PS, and age) was greater than the (PSV of right ICA, and PS).

**Table 3:** ROC prediction curve of each index of coronary heart disease.

Index	AUC	*S.E*	*P*-Value	95% *CI*	Sensitivity	Specificity
PS	0.740	0.034	0.000	0.673–0.807	0.613	0.831
PS + PSV of right ICA	0.762	0.033	0.0000	0.697–0.828	0.754	0.620
PS + PSV of right ICA + Age	0.778	0.030	0.000	0.719–0.847	0.782	0.648

AUC: area under curve; *S.E*: standard deviation; *CI*: Confidence Interval; PS: carotid artery plaque score; PSV: peak systolic velocity; ICA: internal carotid artery.

## 5. Discussion

Our results demonstrate that age and carotid blood flow, combined with PS on carotid ultrasonography, are more predictive for the presence of CHD than PS alone. Ultrasonographic detection of subclinical atherosclerosis improves cardiovascular risk stratification, but uncertainty persists about the most discriminative method to apply.

Yerly and colleagues [28] found that the atherosclerosis burden score (ABS), a novel straightforward ultrasonographic score that sums the number of carotid and femoral arterial bifurcations with plaques, significantly outperformed common carotid intima-media thickness, carotid mean/maximal thickness, and carotid/femoral plaque scores for the detection of coronary artery disease (ROC curve AUC = 0.79; *P* = 0.027 to <0.001 with the other five US endpoints) in 203 patients undergoing coronary angiography. ABS was also correlated with CHD extension (*R* = 0.55; *P* < 0.001).

Morito *et al.* [22] found that PS may more closely represent the atherosclerotic condition of the carotid artery than IMT. Yuk and colleagues [29] showed that both C-IMT and carotid plaque were important predictors of death and MACE, even after adjustment for cardiovascular risk factors, especially in patients with CHD. They also showed that carotid plaque had a higher heart risk than C-IMT in this study group. This suggests that the presence of carotid plaque had greater prognostic significance than C-IMT in this study group.

Carotid hemodynamics is an important independent risk factor for carotid atherosclerosis, as well as CHD [30]. Several studies have confirmed that hemodynamic factors participate in the process of atherosclerosis plaque formation [19,20]. Morito *et al.* showed [22] that increased PS is an independent predictor of the presence of coronary artery disease. Our finding of PS predicting the presence of CHD further supports their finding. In the multivariate analyses, we found variables significantly related to the presence of CHD included PS, PSV of the right ICA and age.

The data in the present study also showed PSV and RI are higher in CHD group than non-CHD group. Weber *et al.* [31] demonstrated that measures of pulsatile arterial hemodynamics may complement Doppler echocardiography for the diagnosis of heart failure with preserved ejection fraction. Terzi *et al.* performed [32] a meta-analysis on 11 population-based studies (54,336 patients). They showed that PS, compared with C-IMT, had a significantly higher diagnostic accuracy for predicting future myocardial infarction events (AUC 0.64 *vs.* 0.61, relative *OR* 1.35; 95% CI 1.1–1.82, *P* = 0.04). A meta-analysis of 27 diagnostic cohort studies (4878 patients) also showed a higher, but non-significant, diagnostic accuracy of PS compared with C-IMT for the detection of CAD (AUC 0.76 *vs.* 0.74, *P* = 0.21 for relative *OR*) [33]. In the present study, the AUC (0.778) of the collective model of carotid ultrasonography was greater than these others.

Our results have several limitations. This is not a population-based study where participants have been selected randomly. Although investigators did not influence the participation (participants voluntarily accept to be screened), the way the study was conducted might represent a potential limitation in the external validity of the results. Furthermore, the diagnostic assessment for hypertension, hyperlipidemia and diabetes was documented with a questionnaire, and no data on prescribed medications or treatment compliance were recorded. In conclusion, the model of PS and PSA of RICA has greater predictive value for CHD than PS alone. Adding age to PS and PSA of RICA further improves predictive value over PS alone.

## 6. Conclusions

In conclusion, the model of PS and PSA of RICA has greater predictive value for CHD than PS alone. Adding age to PS and PSA of RICA further improves predictive value over PS alone.
